# Azacitidine induced lung injury: report and contemporary discussion on diagnosis and management

**DOI:** 10.3389/fonc.2024.1345492

**Published:** 2024-02-09

**Authors:** Ruah Alyamany, Ahmed Alnughmush, Malak Almutlaq, Mohammed Alyamany, Mansour Alfayez

**Affiliations:** ^1^ Department of Hematology, Stem Cell Transplant and Cellular Therapy, Oncology Centre, King Faisal Specialist Hospital and Research Centre, Riyadh, Saudi Arabia; ^2^ Department of Medicine, King Faisal Specialist Hospital and Research Centre, Riyadh, Saudi Arabia; ^3^ College of Medicine, Imam Mohammad Ibn Saud Islamic University, Riyadh, Saudi Arabia

**Keywords:** azacitidine induced lung injury myelodysplastic syndrome, acute myeloid leukemia, azaciditine, hypomethylating agents, acute lung injury, pneumonitis

## Abstract

Azacitidine, a hypomethylating agent, has caused a paradigm shift in the outcomes of patients with myelodysplastic syndrome (MDS) and acute myeloid leukemia (AML) who are not eligible for stem cell transplantation, particularly in combination with BCL2 and IDH inhibitors. Azacitidine and Azacitidine-based combinations have been widely considered a safe low-intensity therapy when compared to traditional conventional treatments. The development of lung toxicity from azacitidine is not a well-characterized adverse event. However, if it happens, it can be fatal, especially if not recognized and treated promptly. In this review, we aim to familiarize the reader with the presentation of azacitidine-induced lung injury, provide our suggested approach to management based on our experience and the current understanding of its mechanism, and review the literature of 20 case reports available on this topic.

## 1 Introduction

Myelodysplastic syndrome (MDS) and acute myeloid leukemia (AML) are clonal hematological diseases with immunological, genetic, and epigenetic heterogenicity. MDS and AML both result in ineffective hematopoiesis and dysmorphism of cells, leading to cytopenias and their complications, including infections and bleeding (1). Additionally, the progressive impairment in growth and differentiation through the hypermethylation of the tumor suppressor gene, such as p15INK4B, can promote disease progression from MDS to AML (2–5).

Epigenetic abnormalities, such as hypermethylation of CpG island, are believed to play an essential role in leukemogenesis, which can lead to the silencing of tumor suppressor genes, which, in turn, drives the neoplastic transformation to AML (3, 6–9). MDS and AML tend to occur more often in people over the age of 60 years, with a male predominance (10, 11). Since most patients are of older age, the chances of having more comorbidities and being unfit increase, which, in turn, limits the ability to use curative therapies, such as stem cell transplant (1).

5-azacytidine, an analog of the pyrimidine nucleoside cytidine, has been studied to treat acute leukemia in the United States since the early 1970s. Clinical trials have primarily focused on patients with an illness resistant to conventional chemotherapy. The findings of these studies showed that 5-azacytidine is effective in treating AML. Following that, clinical trials looked at 5-azacytidine’s impact on other hematological diseases including MDS, non-hematological neoplasms, such as solid tumors, hereditary hematologic diseases, and hemoglobinopathies (thalassemia and sickle cell anemia). The Cancer and Leukemia Group B (CALGB) started a series of clinical trials with 5-azacytidine in MDS patients in 1984. These studies, and other supporting evidence, led to the US FDA approval of 5-azacytidine to treat MDS in May 2004. By blocking DNA methyltransferase, 5-azacytidine prevents the methylation of newly produced DNA (DNMT), restoring normal function to genes involved in differentiation and proliferation (12–16). Since then, azacytidine-based combinations have also been tested and developed in MDS, although with limited success, and in AML, where it led to a number of subsequent US-FDA approvals in patients ineligible for high-intensity therapy (10, 17–24).

Azacitidine is considered to have a relatively safe toxicity profile, with the majority being cytopenia and gastrointestinal symptoms. However, there has been growing evidence of lung toxicity complicating treatment with azacitidine. In our review, we aim to shed light on the manifestations and management of this rare but potentially fatal event and further add to the accumulative knowledge published by providing our experience in managing azacitidine-induced pneumonitis in an AML patient.

## 2 Case report

A 63-year-old male was referred to our institute with a diagnosis of acute leukemia after presenting with fatigue, weight loss, and bruising for two weeks. He was not known to have any past medical illnesses. He had a history of heavy smoking with a pack-year smoking index of 40 but stopped smoking almost five years prior to being diagnosed with leukemia. His initial investigations showed pancytopenia on his complete blood count (CBC) and around 50% blasts on the peripheral blood smear. Bone marrow aspirate and biopsy showed features suggestive of AML, with 56% myeloid blasts detected by flow cytometry. Based on the mentioned investigations along with cytogenetic and molecular testing, he was diagnosed with AML with myelodysplastic-related changes according to the World Health Organization (WHO) classification and stratified as having an adverse-risk disease based on the European Leukemia Network (ELN) risk stratification. The patient refused intensive chemotherapy and stem cell transplant, so he was started on cycle 1 of azacitidine 75 mg/m2 IV for seven days and venetoclax for 21 days. Before starting azacitidine and venetoclax, a high aspergillus galactomannan antigen (14.18) was incidentally found. He was asymptomatic.

**Figure 1:**
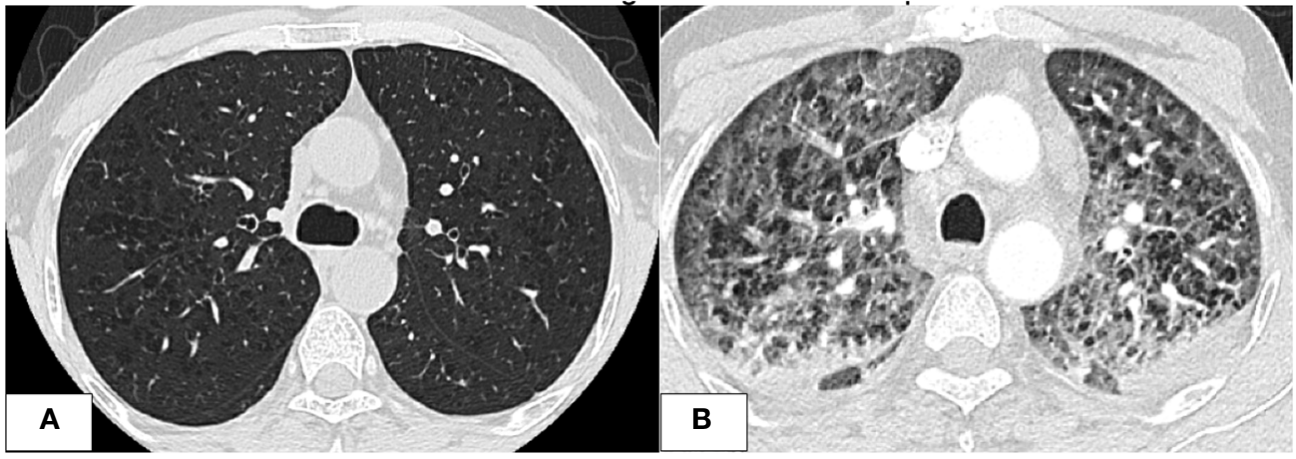
CT chest images. **(A)** After the first cycle of azacitidine, at that time, the patient was treated for invasive fungal infection. Imaging shows significant improvement of previous bilateral ill-defined nodular opacities with residual GGOs and micronodules. **(B)** Imaging was done at the time of the development of respiratory symptoms after the second cycle of azacitidine. Imaging showed worsening diffuse GGOs with smooth interlobular septal thickening and centrilobular nodules with bilateral bronchial wall thickening and small bilateral pleural effusions.

Further investigations with imaging of the chest and paranasal sinuses with computed tomography (CT) were done; the chest CT showed multiple bilateral peri-broncho-vascular rounded central consolidative nodules and cavities representing an inflammatory/infectious process. He was started on Posaconazole. Bronchoscopy and bronchoalveolar lavage (BAL) were negative for infections, including aspergillus galactomannan antigen. However, he was continued on antifungal treatment for six weeks as he was labeled to have a probable invasive fungal infection. While on antifungal treatment, he was started on cycle 1 of azacitidine and venetoclax and achieved remission, and a repeat CT chest after completion of antifungal therapy showed resolution of the previous findings. He initially refused to receive further cycles; however, after three months, he had a relapse with 80% circulating blasts, so he was restarted on azacitidine and venetoclax. On day 2 of the second cycle of azacitidine and venetoclax, he developed a fever and tachypnea. Chest examination was normal, and chest x-ray did not reveal any significant abnormality and had neutropenia on CBC. A microbiological work-up was sent, and he was started on empirical antibiotics with meropenem as he was kept on prophylactic levofloxacin, according to the institute’s febrile neutropenia protocol. Despite empirical antibiotics, he continued to have fever and worsening symptoms. Antimicrobial therapy was upgraded with antifungal treatment, vancomycin, and later sulfamethoxazole-trimethoprim, yet there was no significant improvement. CT chest showed bilateral diffuse ground-glass opacities, smooth interlobular septal thickening, severe emphysematous changes, and small bilateral pleural effusions with no evidence of pulmonary embolism. The patient was kept on empirical antibiotics and completed seven days of azacitidine. On day 11, he became more tachypneic and hypoxic and was transferred to the intensive care unit (ICU) and was started on oxygen therapy with a high-flow nasal cannula. He continued empirical antimicrobial therapy despite all infectious work-ups returning negative (see **Table 1**). A bronchoscopy with bronchoalveolar lavage was done and was negative for malignant cells and infectious causes. BAL fluid was bloody with 24,500 x 106/L of RBCs. He was started on Methylprednisolone 40 mg IV BID for five days for possible drug-induced pneumonitis while continuing antibiotics. He improved clinically, was weaned off oxygen therapy, and was discharged from the hospital. He was re-admitted for the third cycle of azacitidine and venetoclax. On day 5 of cycle 3 of azacitidine, he developed a fever, shortness of breath, and hypoxia. Chest x-ray showed bilateral diffuse reticulonodular opacities. An infectious work-up was sent, and he was started on empirical antimicrobial therapy. CT chest showed diffuse bilateral ground glass densities with smooth interlobular septal thickening, centrilobular nodules, and right upper lobe ground glass opacity with focal subpleural infiltrate (**Figure 1**). Azacitidine-induced pneumonitis was our top differential diagnosis as the symptoms reoccurred with rechallenging with azacitidine, and the Naranjo score was 8, which makes the diagnosis probable (**Table 2**). Azacitidine was discontinued, and he was started on dexamethasone 4 mg IV BID for three days, then tapered down and changed to prednisone orally, which was stopped after six weeks with clinical and radiological improvement. Treatment of his AML was changed to cladribine, low-dose cytarabine with venetoclax with no similar lung toxicity. The patient achieved complete remission, however he refused further treatment, relapsed 4 months later and died within a month of his relapse.

**Table 1:** Investigation results at the time of the development of azacitidine-induced lung injury.

Investigation	Result				
CBC	WBCs	ANC	Eosinophils	Hgb	Plts
	0.41 x10^9^/L	0.06 x10^9^/L	0.01 x10^9^/L	80 g/L	6 x10^9^/L
AFB Culture	Negative				
Blood, respiratory, urine, and throat cultures	Negative				
Fungal culture	Negative				
Aspergillus galactomannan Antigen (serum and alveolar fluid)	Non-reactive				
CMV IgM and viral load	Negative				
Fungitell Qualitative and Quantitative Tests	Negative				
HIV, HTLV 1-2, Hepatitis A, B and C	Negative				
Respiratory PCR Multiplex and Pneumonia Panel (respiratory viruses, atypical bacteria, and other bacteria organisms)	Negative				
COVID-19 PCR	Negative				

**Table 2:** The Naranjo scale results’ classifications (25).

Score	Classification
<0	Doubtful
1 – 4	Possible
5 – 8	Probable
≥ 9	Definite

## 3 Discussion

### 3.1 Azacitidine

#### 3.1.1 Introduction and mechanism of action

Azacitidine, also known as 5-azacitidine, is a ring analog of the pyrimidine nucleoside of cytidine with a nitrogen atom instead of carbon at the fifth position of the heterocyclic ring (3, 26, 27). It is a first-in-class hypomethylating agent. It has a dose-dependent action, which leads to dysregulation of the RNA and DNA.

It works on the RNA by being phosphorylated to azacitidine triphosphate and then incorporated into RNA, inhibiting RNA and disturbing protein synthesis (3). Azacitidine hypomethylates the replicating DNA and inhibits its function through the dephosphorylation of azacitidine to 5-aza-2’ V-deoxycytidine diphosphate by ribonucleotide reductase (6). The 5-aza-2’ V-deoxycytidine diphosphate then is phosphorylated into triphosphate, which binds stoichiometrically to DNA methyltransferase and inhibits it irreversibly which hypomethylates the DNA regulatory sequences and increase gene transcription restoring the normal function of tumor suppressor genes and cell maturation (3, 6, 10, 26–28).

In 1982, azacitidine was used in treating thalassemia as it was found to induce the production of fetal hemoglobin through the hypomethylation of the Y globin suppressor gene (12, 29, 30). Afterwards, it showed an improvement in the survival of AML and high-risk MDS patients who cannot undergo stem cell transplant, which led to its approval in these patients (22, 30–32). Azacitidine can be cytotoxic at high doses, but at lower doses, it results in hypomethylation of DNA and differentiation of cells (33). The usual dosing in MDS/AML is 75 mg/m (2)/day for seven days every four weeks (32, 34, 35).

#### 3.1.2 Adverse effects

In general, medications cause adverse effects in 0.07% of admitted patients and can be fatal in 0.003% (36). One way to assess the correlation between medications and toxicities is using the Naranjo scale, one of the most used tools to detect medications’ toxicities (10). It uses a scored systemic questionnaire, which classifies the possibility of drug-induced toxicity into four categories (**Table 2**) (25). Azacitidine usage in hematological malignancy is considered a low-intensity treatment that is generally well-tolerated and can be administered in the outpatient setting (1). The commonly reported side effects are usually uncritical (**Table 3**). Nevertheless, there has been recent scattered evidence of severe side effects, including lung toxicity, reported in <0.1% of cases (10).

**Table 3:** Azacitidine reported adverse effects.

Hematological system^*^	Anemia, thrombocytopenia, leukopenia and neutropenia, recurrent infections, bleeding, fatigue (37, 38).
Gastrointestinal and hepatobiliary systems^*^	Abdominal pain, constipation, diarrhea, nausea, vomiting and mucositis (3, 10, 23, 37–41).
	Hepatic toxicity (28).
Integumentary and musculoskeletal systems	Injection site reaction. ^**^ (1)
	Bony pain (28).
Renal and genitourinary systems	Renal tubular acidosis (28).
Neurological and psychological systems	Insomnia, weakness, seizures, and coma. ^***^ (3, 10, 23, 28, 39–41)

^*^ Most common adverse effects of azacitidine (37, 38).

^**^ The dose may be divided into three injections to avoid injection-site irritation (1).

^***^ Neurological symptoms are infrequent (28).

### 3.2 Azacitidine-induced lung toxicity

Although azacitidine-induced lung toxicity is rare, it can have dreadful outcomes, which makes early recognition and treatment initiation crucial for reversibility and survival (37). Azacitidine-induced lung toxicity comes in different forms with various presentations (**Tables 4**, **5**). Fever and respiratory symptoms were the most common symptoms reported, commonly mistaken for an infection (42). The reported cases have occurred with the usual dose of azacitidine in MDS/AML, 75 mg/m (2)/day for 5-7 days, and can occur at any time after starting azacitidine (2, 3). Toxicity from azacitidine is irrelevant to its cumulative dose (46). Most cases occurred after eight weeks of initiation, i.e., the second cycle of azacitidine, but others have been widely inconstant (4, 10, 37, 38, 47, 48). The severity of lung injury is highly variable, but in most cases, the longer it takes to recognize it and start steroids, the higher the risk of serious complications, including mortality (49).

**Table 4:** Clinical manifestations of azacitidine-induced lung toxicity (1, 6, 10, 37, 42–44).

Symptoms	Signs and radiological findings
•Fever •Cough (29.5%) •Shortness of breath (29.1%) •Nasopharyngitis (14.5%) •Exertional dyspnea (14.1%) •Productive cough (11.4%) •Rhinorrhea (10%) •Post-nasal drip (5.9%) •Nasal congestion (5.5%) •Hemoptysis (<5%) •Respiratory distress (<5%)	•Hypoxia •Tachypnea •Lung crackles (10.5%) •Wheezing (8.6%) •Decreased breath sounds (7.7%) •Pleural effusion (6.4%) •Rhonci (5.9%) •Atelectasis (5%) •Sinusitis (5%) •Pulmonary infiltrates (<5%) •Pneumonitis (<5%)

**Table 5:** Forms of azacitidine-induced lung toxicity (1, 3, 5, 37, 43, 44).

Interstitial pneumonitis (IP)	
Bronchiolitis obliterans organizing pneumonia (BOOP)	
Cryptogenic organizing pneumonia (COP)^⊕^	
Idiopathic pulmonary fibrosis (IPF)	
Eosinophilic pneumonia (EP)	
Pleural effusions	

^⊕^Organizing pneumonia is characterized by fibroblastic tissue of the alveoli, alveolar ducts, and terminal bronchioles with a plug-like appearance (45).

### 3.3 Pathogenesis of azacitidine-induced lung injury

**Figure 2:**
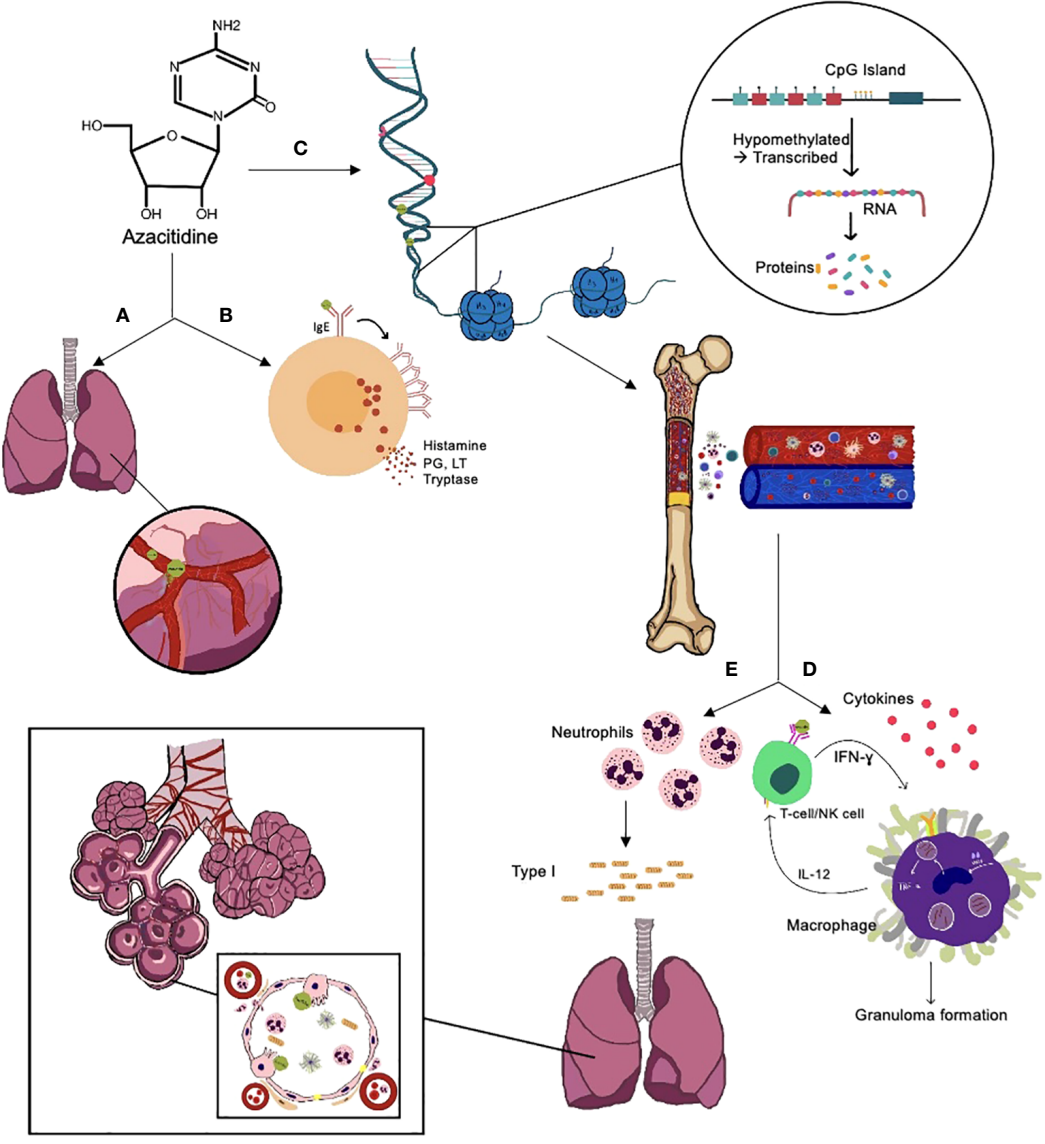
Suggested mechanisms of azacitidine-induced lung injury. **(A)** Direct toxicity to the lungs through damage to the capillary endothelial cells, causing fluid leakage and pulmonary edema, leading to respiratory distress syndrome and interstitial pneumonitis, disturbs the normal production of surfactant. **(B)** Type I hypersensitivity reaction; azacitidine activates the IgE on mast cells, leading to IgE aggregation and release of histamines, leukotrienes (LT), prostaglandins (PG), and tryptase, which lead to vasodilation, increased vascular permeability, and tissue damage. **(C)** Hypomethylates DNA, leading to a recovery in normal hematopoiesis in the bone marrow and immune system recovery. **(D)** Type IV hypersensitivity reaction and azacitidine's ability to augment IFN- ɣ intracellularly, which increases macrophage activation and, as a result, granuloma formation. **(E)** Recovery of hematopoiesis leads to increased neutrophil production, which increases neutrophil elastase and collagen type I production, leading to fibrosis, and can also damage the type II pneumocytes responsible for the repair process.

The underlying mechanism of azacitidine-induced lung injury is not entirely understood. There have been multiple suggested mechanisms (**Figure 2**), including:

(1) Direct cytotoxicity from the drug is similar to gemcitabine-induced toxicity because of the molecular similarities between azacitidine and gemcitabine (5, 6, 30, 43). Gemcitabine damages the capillary endothelial cells, causing fluid leakage and pulmonary edema, leading to respiratory distress syndrome and interstitial pneumonitis (50–53). Also, cytidine analogs can alter the synthesis of surfactants by disturbing the production of essential phospholipids (54). In cases of delayed presentations, i.e., after multiple cycles of azacitidine, toxicity can be related to the cumulative dose of azacitidine (55).
(2) Inflammatory and immune-mediated injury, supported by the findings of lymphocytosis on pleural and alveolar fluid analysis and the improvement following immunosuppressive medications, such as steroids (56). Some of the possible immune-related mechanisms include:
  a. Neutrophil-induced parenchymal injury: in patients with recovered hematopoiesis, neutrophils sequestrate in the lungs and overexpress neutrophil elastase, which increases collagen content and fibrosis (42). This thesis might explain lung injury in patients with recovered counts (43).
  b. Cytokine overexpression through azacitidine’s ability to augment intracellular INF-ɣ increasing macrophage activation and changing the chromatin configuration to increase the transcription of pro-inflammatory genes, helping in tumor control but can cause collateral damage to the surrounding tissues, including the lungs (57, 58). The activation of cytokines, e.g., interleukin-5 (IL-5), by the helper T-cells, triggered by the medication leads to the build-up of eosinophils in the lungs (59, 60).
  c. Azacitidine-induced autophagy; lysosome-dependent degradation of cells resulting in acute and chronic inflammation of the lungs (61). This is particularly true in hypersensitivity pneumonitis (62).
  d. Oxidative stress inhibits the ERK pathway signaling in T-cells (30).
(3) Hypersensitivity reactions (30): both type I and type IV hypersensitivity reactions have been described to be the underlying mechanisms of azacitidine-induced lung injuries.
  a. Type I hypersensitivity reaction; associated with high IgE levels and broncho-centric granuloma as described by Nair et al. (28) This can explain why the toxicity occurs within a few days after the exposure to azacitidine with features of eosinophilic pneumonitis (5, 55). Drug-induced eosinophilic pneumonitis usually happens within the first eight weeks after the drug initiation (28).
  b. Type IV delayed hypersensitivity reaction; this is especially true in the immune reconstitution phase, where CD8+ T-lymphocytes are activated by interleukin-2 (IL-2) and interferon-gamma (IFN- ɣ), which leads to the formation of sarcoid-like granulomas with epithelioid giant cells surrounded by a ring of fibroblasts (30, 47, 63, 64).
(4) Impaired repair by type II pneumocytes (30).
(5) Lineage reprogramming of different cells due to epigenetic priming by azacitidine; however, this theory is questionable (65).
(6) Upregulation of type I collagen synthesis leads to pulmonary fibrosis through the DNA hypomethylation feature of azacitidine (6, 30, 66). However, Parker et al. were unsuccessful in activating type I procollagen genes in the human embryonic lung fibroblasts when they exposed them to azacitidine (67).

The consequence of the different suggested mechanisms is the damage to the alveolar epithelium and disequilibrium of the activity and inhibition of metalloproteinase, leading to intracellular and plasma protein leak and, subsequently, an inflammatory response in the alveolar airspaces, which stimulates repair and promotes fibrosis (45). In cases resulting in organizing pneumonia, further plug formation against the pores of Kohn due to the production of fibro-myxoid material occurs, leading to the characteristic features of organizing pneumonia (68, 69).

### 3.4 Risk factors of azacitidine-induced lung injury

To our knowledge, there is no definite way to predict which patient will develop this toxicity. Nevertheless, upon reviewing the cases described in the literature (**Table 6**), along with our case, we listed some possible factors that might increase the risk of developing lung injury after exposure to azacitidine, which include:

- History of previous or active cigarette smoking (71).
- Previous lung infections (72, 73).
- Underlying airway or parenchymal lung disease.
- Exposure to environmental toxins and medications that have a toxic effect on the lungs (74, 75).
- Chronic gastric-content aspiration (76).
- High neutrophils count (42, 77).
- Leukemic lung infiltration.

**Table 6:** Cases of azacitidine-induced lung injury reported in the literature.

Author	Demographics	Relevant Medical Background	Diagnosis	Presenting Features	Time of onset	Radiological Findings	BAL findings	Pathological findings	Naranjo Score/class	Management	Outcome
Adams et al. (1)	71 y/o M	DLD, refractory anemia, and gout	MDS	Fever, wheezing, and crackles	Cycle 1	Patchy bilateral interstitial opacities with perihilar and alveolar shadowing	•Petechiae •Watery secretions •Negative infectious work-up	•Organizing pneumonitis •Interstitial and alveolar fibrosis •Marked atypia of pneumocytes	Probable	•Empirical antibiotics	Died
Hueser & Patel (70).	55 y/o F	--	MDS	Fever, dyspneia	Cycle 1	Bilateral diffuse interstitial opacity	--	--	Probable	•Oxygen therapy •Methylprednisolone 0.2g/day	Improved
Vasu et al. (44)	65 y/o M	--	MDS	Cough, fever and chills	Cycle 1 of **Decitabine.**	Left lower lobe consolidation	--	Patchy areas of organizing pneumonia with fibrin balls within the alveoli and air spaces	--	•Methylprednisolone	Improved
Hayashi et al. (5)	74 y/o M	Ex-smoker (40-pack-year)	MDS	Fever, dry cough, and dyspnea	Cycle 1	•Non-segmental consolidation with air bronchogram and surrounding GGOs. Mediastinal LN enlargement Pleural effusion.	--	--	Probable	•Empirical antibiotics •Discontinued azacitidine Methylprednisolone 1000 mg/day x3 (started on 31st day after Aza) → oral prednisolone, tapered gradually.	Improved
Kotsianidis et al. (58)	55 y/o M	--	MDS	Fever, hypoxia, hypercapnia	Cycle 1	•Peribronchovascular nodules and consolidations •Multiple centrilobular micronodules •Mild interlobular septae thickening	--	--	Highly suggestive of azacitidine-induced sarcoidosis aggravation	•Prednisolone 0.5 mg/kg/day •Oxygen therapy	Death
Nair et al. (28)*	76 y/o M	Ex-pipe smoker for 3 years, 30 years prior to presentation	MDS	Fever, dyspnea, dry cough, diminished breath sounds and bilateral crackles	Cycle 2	•Diffuse bilateral infiltrates with surrounding GGOs, predominantly in the bases and periphery. •Mediastinal and hilar LN enlargement	Negative for microbiological work-up	•Organizing pneumonia Alveolar fibroblastic plugs •Eosinophilic infiltration (eosinophilic pneumonitis)	--	•Empirical antibiotics •Solumedrol 1 mg/kg IV BID → prednisone and then tapered off over 2 months	Improved
Pillai et al. (42)	74 y/o F	--	MDS	Fever, dyspnea and dry cough	Cycle 2	Bilateral peribronchiolar and reticulonodular shadowing, GGO, pleural effusion. No PE	Negative of microbiological work-up	NA	--	•Empirical antibiotics Discontinued azacitidine •Methylprednisolone 1.5 g/day for 3 days → prednisolone oral	Radiological improvement but remained symptomatic for 9 months.
Sekhri et al. (6)**	56 y/o M	--	MDS	Fever, dyspnea, dry cough and hypoxia	Cycle 2	•Extensive bilateral airspace disease •Many nodular opacities •No PE	--	•Organizing pneumonia •Bronchocentric granuloma •Negative microbiological and connective tissue disease work-up	Probable	•Empirical antibiotics •Discontinued azacitidine and changed to decitabine. •Methylprednisolone Oxygen therapy (mechanical ventilation)	Improved
Kuroda et al. (49)⁂	72 y/o M	CAD	MDS	Fever, dyspnea, productive cough (blood and mucous), and wheezing with hypoxia	Cycle 1	•Interstitial and GGOs bilaterally •Mediastinal LN enlargement •Bilateral pleural effusion	--	NA	Probable	•Empirical antibiotics •Methylprednisolone 500 mg IV x 4 days	Died
Ahrari et al. (4)+	73 y/o M	Mycobacterium fortuitum positive blood culture and he was started on therapy.	MDS	Fever, chills and night sweats	Cycle 3	•Perihilar GGOs and reticulation •Patchy peripheral airspace consolidation •Bilateral hilar LN enlargement	•Negative microbiological work-up •Eosinophilia	NA	Probable	•Empirical antibiotics •High dose of prednisone, tapered over 4 weeks (started after the 8th cycle of azacitidine)	Death
Verriere et al. (38)	86 y/o F	HTN, CAD and smoking	AML	Skin rash, fever, cough, nausea, abdominal, ear pain and weakness	Cycle 3	•Diffuse interstitial and GGOs •Mediastinal and hilar LN enlargement	--	--	--	•Empirical antibiotics •Oxygen therapy •Steroids; 0.75 mg/kg/day •Discontinued azacitidine	Improved
Molina et al. (43)‡	89 y/o F	--	AML-MRC	Dyspnea, fatigue, hypoxia, and bibasilar crackles	Cycle 11	•Subpleural bilateral GGOs •Posterior right lung base honeycombing •Increased interstitial lung markings bilaterally •Increased bronchial wall thickness	--	--	--	•Prednisone 20 mg oral daily for 8 weeks	Improved
Alnimer et al. (3)	67 y/o M	T2DM, HTN and 15-pack-years of cigarette smoking	MDS	Productive cough, hypoxia, bilateral basal fine inspiratory crackles	Cycle 2	•Massive multifocal bilateral pulmonary consolidation and GGOs •Pleural effusion	•Negative for microbiological workup. •Pleural fluid analysis: Transudative with LDH of 206 U/L and 90% lymphocytes and negative cytology for malignant cells.	Chronic non-specific inflammation with macrophages consistent with organizing pneumonia.	Probable	•Empirical antibiotics •Oxygen therapy (non-invasive ventilation) •Methylprednisolone 60 mg IV BID x7 days → 40 mg IV BID x4 days → 20 mg IV BID x3 days → oral prednisolone slowly tapered over 2 weeks	Improved
Makita et al. (2)	77 y/o M	Follicular lymphoma	t-MDS	Fever and hypoxia	Cycle 2	•Bilateral diffuse ground-glass opacities	--	--	--	•Empirical antibiotics •Prednisolone 0.5 mg/kg	Improved
Misra et al. (30)†	67 y/o F	–	MDS	Fever, dyspnea, and dry cough	Cycle 1	•Diffuse bilateral GGOs •Bilateral pleural effusion	--	--	--	•Empirical antibiotics •Corticosteroids at a dose of 1 mg/kg	Improved
Oka et al. (47)	75 y/o F	--	MDS	Dry cough and dyspnea	Cycle 3	Nonsegmental consolidations and GGOs	•Lymphocytosis (34%) •No malignant cells or infectious agents.	Organizing pneumonia and sarcoid-like granulomatous patterns	7 (Probable)	•Empirical antibiotics •Discontinued azacitidine •Methylprednisolone 1000 mg/d x3 days → tapered with oral prednisolone over 6 months	Improved
Nguyen et al. (10)§	75 y/o F	ET on HU, group 2 pulmonary HTN, paroxysmal A-fib on apixaban and diastolic HF	AML	Dyspnea, hypoxia, hemoptysis, and hypotension	Cycle 1	•Diffuse GGOs/consolidative changes •Bilateral pleural effusion	•Bilateral lower lobes mucosal thickening and friability •Infectious work-up was negative •BAL showed 87% lymphocytes	--	--	•Empirical antibiotics •Prednisone 30 mg 2 times per day, tapered off over 5 weeks	Improved
Litvin et al. (37)	70 y/o M	COPD, lipoid pneumonia, and latent TB	AML	Fever, tachycardia, tachypnea, and hypoxia	Cycle 1	•Patchy bilateral GGOs with atelectasis •Bilateral pleural effusion	•170 RB cells/mm3 and 10 WB cells/mm3 •Negative infectious work-up	--	--	•Empirical antibiotics •Discontinued azacitidine •Oxygen therapy Methylprednisolone 1 mg/kg	Improved
Cabral et al. (56)	56 y/o M	Ex-smoker (40-unit-pack-year), history of addiction to cocaine, heroin, and cannabis, HTN, T2DM, and DLD	MDS progressed to AML	Dyspnea, fever, and hypoxia	Cycle 2	Diffuse parenchymal ground-glass densification with an NSIP-like pattern.	•320,000 cells/mL, 93% lymphocytes •Negative microbiological work-up	--	7 (Probable)	Empirical antibiotics Methylprednisolone 500 mg x3 → prednisolone 0.75 mg/kg in a slow tapering scheme	Improved
Hutchinson et al. (55)	73 y/o F	Stasis dermatitis, nummular eczema, rosacea, previously resected squamous cell carcinoma of the nose and scalp, sciatica, sweet syndrome, and IBS	ET → JAK2 +ve MDS	Fever, chills and rigors, productive cough and right-sided pleural chest pain, tachycardia, bilateral basilar crackles	Cycle 23	•Bilateral subpleural irregular nodules •Ground glass halos •Right pleural effusion	Negative for infectious work-up	•Inflammatory cells and minimal T-lymphocytes •No neoplastic cells •H&E stain: polypoid fibroblastic aggregations in alveolar sacs + reactive changes in the alveolar epithelium	--	•Empirical antibiotics •Prednisone 50 mg oral daily, gradually tapered off. Azacitidine was replaced with decitabine-cedazuridine	Improved

*IgE levels were elevated at 10,954 IU/mL (normal <200 IU/mL) (28).

**Tolerated decitabine well without developing respiratory complications (6).

The surfactant protein (SP-A) levels were elevated. Steroids were started on day 11 of azacitidine with initial improvement, but later his condition deteriorated (49).

+He received a total of 8 cycles of azacitidine because the presentation was initially attributed to M. fortuitum, but the presentation was later proven to be related to azacitidine after the clearance from M. fortuitum was established, and the patient's respiratory condition continued to worsen. The patient died five months after stopping azacitidine; his death could be attributed to the delay in discontinuing azacitidine, which led to extensive damage to the lung tissue, which could not have been reversed when azacitidine was stopped, and steroids were initiated (4).

‡Patient received decitabine 20 mg/m^2^ IV for five days every 28 days. Investigations were positive for autoimmune antibodies (ANA 1:40, p-ANCA Ab 1:16). After being treated with steroids, she was put back on decitabine without complications (43).

†Initially, the patient improved but later developed pleural effusion (30).

§CMV IgM and IgG and aspergillus antigen were mildly elevated (10).

### 3.5 Differential diagnoses

Multiple diseases have similar features to azacitidine-induced lung injury and need to be considered when patients develop the features of azacitidine-induced lung injury mentioned above. Examples of these differentials include:

▪ Infections are one of the most critical differentials to consider, as it is a common cause of morbidity and mortality in this group of patients and have very similar features to azacitidine-induced lung injury (10). The diagnosis of azacitidine-induced lung injury is commonly delayed because infections have a similar presentation, and the diagnosis of chemotherapy-induced pulmonary toxicity is less common and requires the exclusion of infections (37, 78, 79).
▪ Malignancy, i.e. leukemic infiltrates. In these cases, it is helpful to do a bone marrow examination to evaluate the disease status and, as needed, further diagnostic studies, for example, a lung biopsy (5, 38, 78).
▪ Autoimmune diseases and vasculitis with lung involvement (2, 28, 37, 80).
▪ Extramedullary hematopoiesis.
▪ Sweet syndrome, hyper-eosinophilic syndrome, and pulmonary alveolar proteinosis (28, 38).
▪ Pulmonary hemorrhage, particularly in the setting of low platelet counts or anticoagulation/antiplatelet therapy (10).
▪ Cardiac-related pulmonary edema, i.e., heart failure (10, 78).
▪ Other medications or interventions that can cause lung toxicity (**Table 7**).
▪ Exacerbation of airway diseases or interstitial lung diseases (28).

**Table 7:** Interventions that can cause lung injury similar to azacitidine-induced lung toxicity.

Medication (28, 55, 81–84)	•All-trans retinoic acid •Cytarabine •Daunorubicin •Mercaptopurine •Methotrexate •Etoposide •Rituximab •Cyclophosphamide •Gemcitabine •Fludarabine •Bleomycin •Melphalan •Busulphan •Carmustine •Thalidomide •Amiodarone •Nitrofurantoin •Apixaban
Radiation therapy (80)	
Herbal remedies (28)	

### 3.6 Investigations and diagnostics

The diagnosis of azacitidine-induced lung injury can be challenging and requires a high index of suspicion. The pillars of the diagnosis mainly rely on the clinical assessment and ruling out other causes. The diagnosis can be delayed and is usually considered after no significant improvement following the start of empirical antibiotics and when no other cause is identified (2).

Following are the main navigating steps, we believe, that help reach the diagnosis of azacitidine-induced lung injury:

(1) Detailed history and physical examination. This is the first and one of the most crucial steps in the diagnosis process. It can establish the causative relationship between azacitidine and lung toxicity and helps rule out other causes (59). Some crucial aspects to focus on while attaining the clinical assessment include, but are not limited to:
  ✓ Detailed presenting illness history.
  ✓ Details on exposure history to azacitidine or other cytidine analogues and previous complications.
  ✓ History of lung, cardiac or autoimmune/rheumatological diseases.
  ✓ Smoking exposure; active or previous history of smoking, pack-years, type of smoking, and complications of smoking; many of the patients were either current or previous smokers.
  ✓ History of allergies.
  ✓ Medications and previous therapy exposure (**Table 7**).
  ✓ Occupation, environmental exposures, and use of herbal remedies.
  ✓ Traveling history.
  ✓ History of exposure to TB.
  ✓ History of malignancies
  ✓ Assessing for features of autoimmune diseases and allergic reactions.
  ✓ Careful physical examination.
(2) Diagnostic tests. Azacitidine-induced lung injury is a diagnosis of exclusion (10, 30, 56). Some of the most important investigations to be considered to exclude other causes and further support the diagnosis are listed in **Table 8**.

**Table 8:** Diagnostic tools that can help establish the diagnosis of azacitidine-induced lung injury.

Type of study	Components	Findings suggestive of azacitidine-induced lung injury
Serum and blood investigations	Complete blood count with white blood cell differentials*	Cytopenia is usually described, but can be normal.
	Chemistry	–
	Blood gas	Respiratory alkalosis (3)
	Immunoglobulin levels; IgE especially (28).	IgE can be elevated, especially if following a hypersensitivity pattern.
	Peripheral blood smear	–
	Autoimmune antibodies	Should be negative, but has been reported in 1 case to be positive without other features of autoimmunity
	Cardiac enzymes; CK, Troponin and Pro-BNP	Unremarkable
Microbiological and infectious work-up	Bacterial and fungal cultures from blood, sputum, alveolar/pleural fluid and any other possible sites PJP work-up Viral work-up; CMV, EBV, HSV, and VZV along with other common causes of viral pneumonitis. TB testing	Negative
Imaging (5, 30, 37, 38, 43, 85)	Chest x-rays	Diffuse bilateral interstitial GGOs, patchy infiltrates and consolidations, and alveolar-pattern shadows with or without pleural effusion. Subpleural irregular nodules and mediastinal/hilar lymphadenopathy have also been described.
	Chest CT scan with and without contrast; to rule out pulmonary embolism**	
	Echocardiogram	Unremarkable (3, 6)
	PET-CT if the possibility of extramedullary leukemic infiltrates or other malignancies is considered	Unremarkable
Pathology and other investigations (30, 37, 38)	Bronchoscopy and bronchoalveolar lavage***	Predominant lymphocytosis, with or without eosinophilia. Negative microbiological work-up.
	Lung parenchymal or pleural biopsy. **** (5, 43, 45, 49, 59, 74, 86)	Non-specific changes: diffuse alveolar damage with honeycombing, bronchogenic granulomatous pattern with focal intra-alveolar inflammation and necrosis, fibroblastic tissue in the distal airspaces and interstitial inflammation. Negative microbiological work-up.
	Bone marrow aspirate and biopsy to assess leukemia status	Confirmation of resolution/improvement of hematological disease.
	Electrocardiogram (EKG)	Unremarkable (3).

*Complete blood counts are important to assess to check the blasts, neutrophils, platelets, and eosinophil counts (43).

**In cases with eosinophilic pneumonia resulting from azacitidine-induced lung injury, changes are usually seen in a peripheral distribution in the middle and upper lung zones.4 (4) Pleural effusion was seen in some cases (30, 37).

***Pleural fluid analysis described in the literature was extremely variable.3 (3) Bronchoscopy and bronchoalveolar lavage (BAL) fluid analysis is usually suggestive of active, non-infectious inflammatory conditions, with predominant lymphocytosis and, in some cases, eosinophilia with no identified pathogens (4, 28, 37, 87).

****Lung biopsy can greatly help distinguish azacitidine-induced lung injury from other causes, especially infections. However, biopsies are not always attainable because most cases would have low blood cell counts, especially platelets, after receiving azacitidine or secondary to the unhealthy bone marrow secondary to the original disease, which would put patients at high risk of bleeding with such procedures (2, 49). The pathological features are not highly specific to azacitidine-induced lung injury and can have patterns of different interstitial lung diseases. Some of the described pathological features include diffuse alveolar damage with honeycombing, bronchogenic granulomatous pattern with focal intra-alveolar inflammation and necrosis, fibroblastic tissue in the distal airspaces, and interstitial inflammation (5, 43, 45, 49, 59, 74, 86).

Because azacitidine-induced lung injury is a diagnosis of exclusion, recognition and management is usually delayed, which subsequently causes progression to respiratory failure and significant lung damage (28, 49). We suggest using criteria to help increase the suspicion index and possibly establish the diagnosis in a timely manner, especially in cases where obtaining a biopsy is not feasible. We suggest having 8/10 of the following factors of the criteria present to consider the diagnosis of azacitidine-induced lung injury:

(1) Fever and respiratory symptoms mimicking pneumonia in patients with AML or MDS who have been exposed to azacitidine (2, 4, 28).
(2) No improvement within 48-36 hours of the use of empirical antibiotics following local guidelines and according to suspected infection (2).
(3) Negative extensive microbiological investigations (2). In some cases, coincidental findings of positive cultures can be confusing and delay diagnosing and treating azacitidine-induced lung injury, which can have dreadful ramifications (4).
(4) Other causes of lung injury are ruled out, including infections, malignancy, exacerbation of underlying lung disease, and other medications or toxins (5, 55).
(5) Clinical, imaging, and pathological patterns in compliance with previous features described features in the literature of azacitidine-induced lung injury (**Table 8**) (5, 55).
(6) Histopathological proof of no active infectious cause.
(7) Naranjo score ≥ 5 (probable-definite) (6).
(8) Clinical improvement following discontinuation of azacitidine (5, 30). Radiological improvement might be slower.
(9) Favorable clinical and radiological response to early initiation of steroids (55).
(10) Recurrence of features upon reintroduction of azacitidine (5, 30, 37, 49). In cases where azacitidine-induced lung injury remains questionable, the benefits of re-introducing azacitidine outweigh the risks. Suppose similar features of lung toxicity recur with the reintroduction. In that case, this can be a distinguishing feature to support the diagnosis and lead to permanent discontinuation of azacitidine and use of alternative medications (2, 37).

### 3.7 Treatment and outcomes

The optimal management remains to be discovered. However, the main treatment strategy established in most reported cases is the clampdown of the inflammatory response, usually established by using corticosteroids (1, 2, 88). The data available needs to be more comprehensive to establish which specific agent is superior in management. Corticosteroids are the most used category of drugs. Other less commonly used agents in interstitial lung diseases include other immunosuppressive medications, cytotoxic agents, cyclophosphamide, and antifibrotic agents, alone or combined. However, there has not been enough evidence for their use in azacitidine-induced lung injury (1, 38, 88).

The timely introduction of steroids and the discontinuation of azacitidine have led to the reversibility of the lung injury, both clinically and radiologically, within days (10, 37). The type of corticosteroid and dosing has yet to be unified, but most cases used high-dose steroids with a slow tapering plan (**Table 6**) (2). Further studies are needed to establish the optimal type, dosing, and tapering plan in such cases (2).

**Figure 3:**
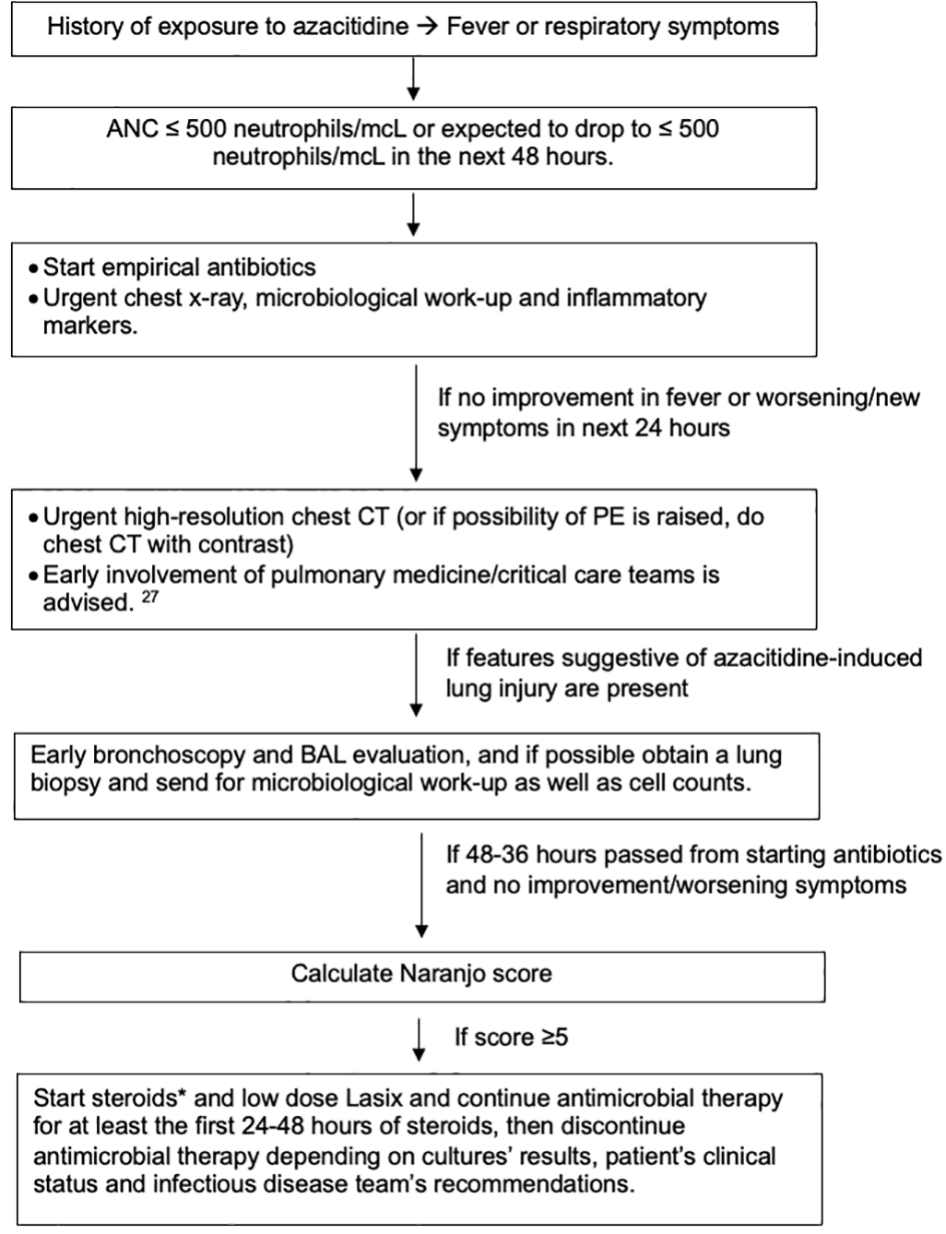
Suggested algorithm for managing azacitidine-induced lung injury. *Steroids type and dose depends on availability, severity of illness and comorbidities that can limit the use of steroids. We generally recommend 1mg/kg as starting dose. Gradual tapering of steroids is preferred if feasible. While patient is on steroids, especially if will be on it for a prolonged time, prophylaxis against pneumocystis jiroveci pneumonia (PJP) and fungal infections, along with protein-pump inhibitors (PPIs) as prophylaxis against gastric ulcers if indicated and vitamin D replacement.

It is reasonable to be hesitant with the use of steroids or other immunosuppressive medications in this scenario, especially early during the presentation, because of the immunocompromised state of the patients and the mimicking picture of other more common infectious causes (2, 42). We must acknowledge the critical consequences of delaying management, with mortality reaching 19% (10, 38). For that reason, we endeavored to create an algorithm that might help ease this difficult decision (**Figure 3**).

In cases of azacitidine-induced lung injury, recurrence of the toxicity can occur with the reintroduction of azacitidine, such as the scenario in our case (37, 49). For that reason, if the possibility of azacitidine-induced lung injury is high, it is better to avoid the use of azacitidine and consider alternative medications, especially if the risk does not outweigh the benefit (37). Sekhri et al. suggested the use of decitabine as an alternative to azacitidine, as it did not cause lung toxicity and allowed for the continuation of therapy (6). Nevertheless, there have been reported cases of lung injury with the use of decitabine, which would make us extra cautious if decitabine is used as an alternative, and close monitoring with early discontinuation of the drug if respiratory symptoms develop is needed (6, 43).

## 4 Conclusion

Azacitidine-induced lung injury is uncommon, occurring in <0.1% of patients (37), but can have terminal outcomes. This makes it an important differential diagnosis when dealing with unexplained fever and respiratory symptoms after exposure to azacitidine. A vigilant evaluation and well-timed management are needed to establish the diagnosis, undo the injury, and prevent atrocious outcomes from happening (30). Several attempts to understand the underlying mechanism have been undertaken, but this remains an area that needs further tackling to establish the predictive factors before starting azacitidine and optimizing the management (38, 49).

## Author contributions

RA: Conceptualization, Data curation, Formal analysis, Investigation, Methodology, Writing – original draft, Writing – review & editing. AA: Writing – review & editing. MAlm: Writing – review & editing. MAly: Writing – review & editing. MAlf: Supervision, Writing – review & editing.

## Glossary

**Table:**

AFB	Acid-fast bacilli
A-fib	Atrial fibrillation
AML	Acute myeloid leukemia
ANC	Absolute neutrophil count
BAL	Bronchoalveolar lavage
CAD	Coronary artery disease
COPD	Chronic obstructive pulmonary disease
CK	Creatinine kinase
CMV	Cytomegalovirus
CT	Chest tomography
CXR	Chest X-ray
DLD	Dyslipidemia
DNA	Deoxyribonucleic acid
ET	Essential Thrombocytosis
GGO	Ground-glass opacities
HF	Heart failure
Hgb	Hemoglobin
HIV	Human immunodeficiency virus
HRCT	High-resolution CT
HTLV	Human T-lymphotropic virus
HTN	Hypertension
HU	Hydroxyurea
IFN	Interferon
IL	Interleukins
LN	Lymph node
MDS	Myelodysplastic syndrome
PCR	Polymerase chain reaction
PE	Pulmonary embolism
PET-CT	Positron Emission Tomography – CT
PG	Prostaglandins
Plts	Platelets
Pro-BNP	Pro-Brain natriuretic peptide
RNA	Ribonucleic acid
TB	Tuberculosis
T2DM	Type 2 diabetes mellitus
WBC	White blood count
